# Electro-Acupuncture Improve the Early Pattern Separation in Alzheimer’s Disease Mice *via* Basal Forebrain-Hippocampus Cholinergic Neural Circuit

**DOI:** 10.3389/fnagi.2021.770948

**Published:** 2022-02-02

**Authors:** Long Li, Jianhong Li, Yaling Dai, Minguang Yang, Shengxiang Liang, Zhifu Wang, Weilin Liu, Lidian Chen, Jing Tao

**Affiliations:** ^1^Rehabilitation Medical Technology Joint National Local Engineering Research Center, Fujian University of Traditional Chinese Medicine, Fuzhou, China; ^2^College of Traditional Chinese Medicine, Chongqing Medical University, Chongqing, China; ^3^TCM Rehabilitation Research Center of SATCM, Fujian University of Traditional Chinese Medicine, Fuzhou, China; ^4^College of Integrated Traditional Chinese and Western Medicine, Fujian University of Traditional Chinese Medicine, Fuzhou, China; ^5^College of Rehabilitation Medicine, Fujian University of Traditional Chinese Medicine, Fuzhou, China

**Keywords:** pattern separation, Alzheimer’s disease, electro-acupuncture, cholinergic neural circuit, chemogenetic technology

## Abstract

**Objectives:**

To explore the effect of electro-acupuncture (EA) treatment on pattern separation and investigate the neural circuit mechanism involved in five familial mutations (5 × FAD) mice.

**Methods:**

Five familial mutations mice were treated with EA at Baihui (DU20) and Shenting (DU24) acupoints for 30 min each, lasting for 4 weeks. Cognitive-behavioral tests were performed to evaluate the effects of EA treatment on cognitive functions. ^1^H-MRS, Nissl staining, immunohistochemistry, and immunofluorescence were performed to examine the cholinergic system alteration. Thioflavin S staining and 6E10 immunofluorescence were performed to detect the amyloid-β (Aβ). Furthermore, hM4Di designer receptors exclusively activated by designer drugs (DREADDs) virus and long-term clozapine-N-oxide injection were used to inhibit the medial septal and vertical limb of the diagonal band and dentate gyrus (MS/VDB-DG) cholinergic neural circuit. Cognitive-behavioral tests and immunofluorescence were performed to investigate the cholinergic neural circuit mechanism of EA treatment improving cognition in 5 × FAD mice.

**Results:**

Electro-acupuncture treatment significantly improved spatial recognition memory and pattern separation impairment, regulated cholinergic system via reduction neuron loss, upregulation of choline/creatine, choline acetyltransferase, vesicular acetylcholine transporter, and downregulation of enzyme acetylcholinesterase in 5 × FAD mice. Aβ deposition was reduced after EA treatment. Subsequently, the monosynaptic hM4Di DREADDs virus tracing and inhibiting strategy showed that EA treatment activates the MS/VDB-DG cholinergic neural circuit to improve the early pattern separation. In addition, EA treatment activates this circuit to upregulating M1 receptors positive cells and promoting hippocampal neurogenesis in the dentate gyrus (DG).

**Conclusion:**

Electro-acupuncture could improve the early pattern separation impairment by activating the MS/VDB-DG cholinergic neural circuit in 5 × FAD mice, which was related to the regulation of the cholinergic system and the promotion of neurogenesis by EA treatment.

## Introduction

Alzheimer’s disease (AD) is the most common age-related neurodegenerative disease characterized by gradual cognitive impairment. Pattern separation is a fundamental hippocampal process thought to be critical for reducing potential interference among similar memory representations to enhance memory accuracy (Frank et al., 2020). Pattern separation may become less efficient as a result of normal aging, possibly due to age-related changes in the hippocampus-subareas which is a sensitive marker of memory changes (Cayco-Gajic and Silver, 2019; Stevenson et al., 2020). Further research showed that spatial pattern separation is impaired in the early AD stage (Parizkova et al., 2020). Therefore, it is necessary to improve the pattern separation impairment and avoid further damage of cognitive deficits in the early stage of AD.

Electro-acupuncture (EA) is a complementary alternative medicine therapy, which is accepted by the World Health Organization and National Institutes of Health. It uses the acupuncture method by applying an electrical current to acupuncture needles. The effectiveness of EA for cognitive improvement in AD has been proven in various clinical and animal studies (Park et al., 2017; Wang et al., 2020). Our previous studies found that EA treatment could ameliorate the ability of 12-month-old mice with AD to recognize novel objects and familiar objects which was accompanied by increasing neural activity in the hippocampus (Liu et al., 2017). However, it is still unknown whether EA treatment could improve the pattern separation impairment in the early stage of AD.

The dentate gyrus (DG) of the hippocampus is essential to the pattern separation. In various AD model mice, it has been shown that adult hippocampal neurogenesis is impaired (Zeng et al., 2016; Richetin et al., 2017; Zaletel et al., 2018). The mice with AD showed a significant pattern separation impairment in touchscreen-based tasks, whereas the classical Morris water maze test revealed no difference between groups in the early stage of AD (Saifullah et al., 2020). These behavioral changes were associated with adult neurogenesis impaired in DG. The increase of adult hippocampal neurogenesis is efficient in differentiating between overlapping contextual representations to improve the pattern separation impairment (Sahay et al., 2011). Pieces of research indicate that cognitive function improvement by EA is related to promoting neurogenesis (Wang et al., 2014; Yu et al., 2020). Furthermore, EA stimulation could improve cognitive impairment and promote neurogenesis in AD model mice (Li et al., 2014).

The DG is innervated by cholinergic neurons in the basal forebrain. Furthermore, loss of the cholinergic function is associated with decreased synthesis of acetylcholine (ACh) in the medial septum (MS) or the vertical limb of the diagonal band of Broca (VDB) of the basal forebrain (Yan et al., 2018), which contributes to memory deficits in AD (Sultzer, 2018). Disordered regulation of the synthesis, storage, transportation, or degradation of ACh can all result in cognitive impairment (Ferreira-Vieira et al., 2016). Thus, ACh supplementation therapy has been proposed as a treatment for AD. Activation of choline acetyltransferase (ChAT) neurons in the basal forebrain with theta burst stimulation that alleviates the decay in cholinergic synaptic transmission effectively protects against spatial pattern separation impairment in the mice with AD, and this protection was completely abolished by inhibiting the hippocampus newly generated immature neuron survival (Zhu et al., 2017). Accumulating research has reported that EA alters ACh levels via modulation of its metabolism, including increasing the activities of cholinergic receptors, ACh, ChAT, and vesicular acetylcholine transporter (VAChT), and decreasing the activity of enzyme acetylcholinesterase (AChE), thus improving cognitive impairments (Lee et al., 2014; Han et al., 2018). However, it remains to be seen whether EA treatment could improve pattern separation impairment via the basal forebrain-hippocampus cholinergic neural circuit.

Therefore, the present study sought to assess the effectiveness of EA treatment on pattern separation and explore the role of the basal forebrain-hippocampus cholinergic neural circuit in the early stage of AD. We first used four cognitive-behavioral tests to assess the effects of EA treatment on cognitive impairments and cholinergic function in 3-4 months five familial mutation (5 × FAD) mice. Next, we injected an adeno-associated virus with ChAT-driven chemogenetics to inhibit the basal forebrain-hippocampus circuit, clarifying whether EA treatment could promote hippocampal neurogenesis to improve cognitive impairments via this neural circuit.

## Materials and Methods

### Animals

The 5 × FAD mouse (stock #34848, Qianbi Biotechnology Co., Ltd., China) overexpresses amyloid β protein precursor (APP) with K670N/M671L (Swedish mutation), I716V (Florida mutation), and V717I (London mutation), and PS1 with M146L and L286V mutations. Female C57BL/6 wild-type mice (WT) and male 5 × FAD mice were bred, and the offspring genotyping was performed by polymerase chain reaction analysis of tail DNA. Both male and female mice were used in this study, and they were housed in same-sex of 3–5/cage under a 12-h light-dark cycle (lights on at 8:00 am) with *ad libitum* access to food and water at a consistent ambient temperature (21 ± 1°C). All experimental protocols and animal handling procedures were conducted according to the Guide for the Care and Use of Laboratory Animals published by the National Institutes of Health. This study was approved by the Ethical Committee on Animal Experimentation, Fujian University of Traditional Chinese Medicine (FJTCMIACUC2019031).

### Experiment Design

**FIGURE 1:**
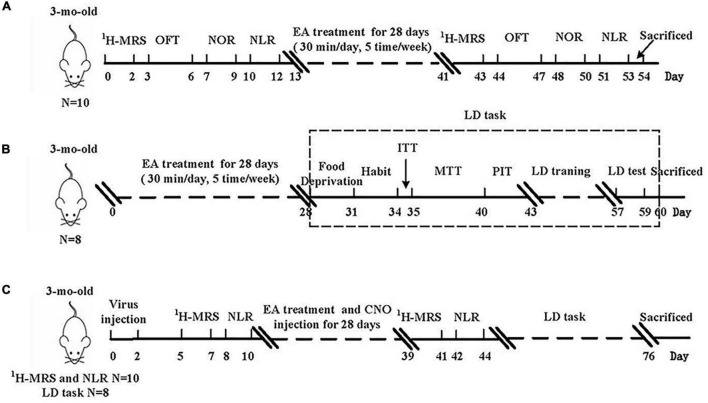
Experimental design of this study. **(A,B)** The experimental procedure of test with EA treatment. **(C)** The experimental procedure shows a strategy for EA treatment and hM4Di DREADDs inhibited. EA, electro-acupuncture; ^1^H-MRS, PRESS-^1^H Magnetic resonance spectroscopy; OFT, open field test; NOR, novel object recognition; NLR, novel location recognition; ITT, initial touch training; MTT, must touch training; PIT, punish incorrect training; LD, location discrimination.

This study was divided into two parts. First (Figures 1A,B), to assess the improvement of cognitive-behavioral tests and cholinergic function in the early stage of AD, 3-month-old 5 × FAD mice were divided into three groups (*n* = 18/group): (i) 5 × FAD group, (ii) 5 × FAD mice that received electro-stimulation at DU20 and DU24 acupoints treatment group (5 × FAD + EA), and (iii) 5 × FAD mice that received electro-stimulation at non-acupoint treatment group (5 × FAD + NA). Moreover, age and sex-matched littermate WT mice were classified as the WT group. Since different cognitive-behavioral tests may interfere with the cognitive function of the mice, each group of mice was divided into two subgroups in the first part. Mice were evaluated for cognitive behavioral tests, including open field test (OFT), novel object recognition (NOR), novel location recognition (NLR), and location discrimination (LD). Choline (Cho) and N-acetylaspartate (NAA) content were detected by PRESS-^1^H Magnetic resonance spectroscopy (^1^H-MRS). Then, mice were sacrificed, and brain slices were used to measurement of amyloid-β (Aβ) plaque and cholinergic pathway biomarkers level.

Second (Figure 1C), to explore the role of the basal forebrain-hippocampus circuit in improving cognitive impairments, 3-month-old 5 × FAD mice were divided into five groups (*n* = 10/group): (i) 5 × FAD group, (ii) 5 × FAD + EA group, (iii) 5 × FAD mice that were injected with hM4Di virus and clozapine-N-oxide (CNO) (5 × FAD + hM4Di group), (iv) 5 × FAD mice that received EA treatment were injected with hM4Di virus and CNO (5 × FAD + EA + hM4Di group), and (v) 5 × FAD mice that received EA treatment were injected with control virus and CNO (5 × FAD + EA + Con group). Moreover, age and sex-matched littermate WT mice were classified as the WT group. Since the results from the first series showed that spatial recognition memory and pattern separation impairment were in the early stage of AD, NLR and LD were used in this second part of the experiments, and all mice were evaluated for cognitive behavioral tests and ^1^H-MRS. Then, mice were sacrificed, and brain slices were used to observe the cholinergic neural circuit projection and expression of hM4Di/Con viruses by confocal (LSM 710, Carl Zeiss, Germany). Next, we were to measure the cholinergic receptors expression and neurogenesis.

### Electro-Stimulation Treatment

The electro-stimulation (EA) or non-acupoint (NA) treatment continued for 28 days (2/20 Hz, 1 mA, 30 min/day, 5 times/week). Mice were awake during treatment. The mouse was restricted to movement and fixed in a net bag to maintain posture by an operator. According to a previous study (Huang et al., 2019), the needle was inserted into the Baihui (DU20) and Shenting (DU24) acupoints in EA group, or non-acupoint under the hypochondrium (the area below the costal region, 2 cm superior to the posterior superior iliac spine and ∼3 cm lateral to the spine) in NA group at a depth of 4–5 mm, and stimulation was generated with an EA instrument (G6805; SMIF, Shanghai, China). Mice in the WT group, 5 × FAD group, and 5 × FAD + hM4Di group were grabbed and fixed under the same conditions without treatment.

### Cognitive Behavioral Tests

Mice were tested in a battery of cognitive-behavioral tests to evaluate cognitive impairments and possible alterations due to EA treatment in the early stage of AD. OFT, NOR, and NLR were conducted pre and post-treatment, and LD was conducted only during post-treatment. Mice were handled in the experimental room for 10 min/day for 3 days prior to the first exposure to the behavioral apparatus. After each mouse, the apparatus and objects were cleaned with a solution of 75% ethanol. All behavioral tests were conducted from 8:00 am to 6:00 pm.

#### Open Field Test

Open field test was used to assess locomotor activity and exploratory behavior (Jawhar et al., 2012). The open field box was made of the white plank with 40 cm × 40 cm and 50 cm high walls divided into peripheral and central. The mouse was singly placed in the center and allowed to move freely to habituate the arena for 10 min on the day before the test. Twenty-four hours later, the behavioral parameter was registered during 10 min and monitoring was done by an automated tracking system (Supermaze, Softmaze, China). The variables recorded were (i) total traveled distance, (ii) average speed, and (iii) time spent in the central part.

#### Novel Object Recognition

**FIGURE 2:**
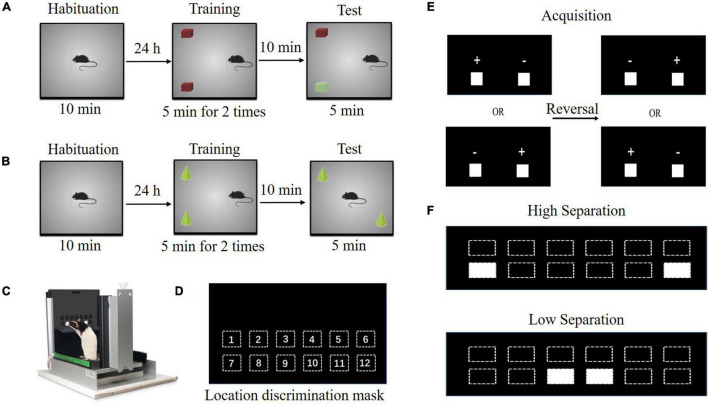
Schematic of cognitive-behavioral tests. **(A)** The paradigms of novel object recognition. **(B)** The paradigms of novel location recognition. **(C–F)** The paradigms of location discrimination (LD). **(D)** The mask of LD with 12 locations is depicted. **(E)** The LD training phase is shown with two response windows illuminated at medium separation. If a mouse makes seven correct responses in eight consecutive trials, the reward-location contingency is reversed**. (F)** Two difficulty levels are depicted in the LD probe.

Novel object recognition and NLR protocols used to measure the object and spatial recognition memory were conducted as described previously (Phan et al., 2015). NOR consists of two training sessions and one test session, each 5 min in duration, separated by 5 min intervals. During the training session, we placed mice in an arena that contained two identical objects for 5 min (Figure 2A). The mice that did not explore objects for 20 s within the 5 min period were excluded from further experiments. We defined exploration as a mouse approaching its nose within 2 cm of the object. This approaching was associated with looking, sniffing, or touching. The test session was done 5 min after training. In this trial, we replaced one of the objects presented in the first trial with a novel object. We then placed mice back in the arena for 5 min and recorded the total time spent exploring each object. Time spent actively exploring of the familiar (F) and novel (N) objects during the test trial were calculated. Novel recognition memory was evaluated via percent investigation for each mouse with a formula [N/(N + F)]*100%. It reflected the time exploring the novel object and the total time exploring both objects.

#### Novel Location Recognition

To test object location memory (Figure 2B), each mouse was placed in the apparatus and was allowed to explore two identical objects for 5 min for two consecutive sessions, separated by 5 min intervals. One of the objects previously presented in the training session was repositioned in the test trial. Time spent active exploring the non-relocated (NR) and repositioned (R) objects were calculated. Location recognition memory was evaluated via percent investigation for each mouse with a formula [R/(NR + R)]*100%. It reflected the time exploring the novel location and the total time exploring both locations.

#### Location Discrimination Task

The location discrimination task protocol used to measure pattern separation was conducted as previously described (Oomen et al., 2013). The touchscreen operant chambers (Bussey−Saksida Mouse Touchscreen Chamber Model 80614, Lafayette Instrument Co., Lafayette, IN, United States) consisted of black perspex walls and a perforated metal floor creating an animal working area (Figure 2C). Each chamber was equipped with a touchscreen at the large base, liquid reward dispenser at the small base, house light, reward chamber light, tone generator, and infrared beams with an observation system. A black plastic mask (28 cm wide × 18 cm tall) was inserted in front of the touchscreen, allowing for different visual stimuli and different tasks to be utilized. The 6 × 2 mask used for the LD task consists of two rows with six windows with stimuli only being presented in the bottom row (Figure 2D). A peristaltic precisely delivered liquid reward, which consisted of 7 μl strawberry milk to entice the mice to perform.

##### Pre-training

All mice were maintained at 85–90% free-feeding body weight throughout behavioral training and testing. Milk was dispensed into the reward magazine prior to placing mice into the chambers before the start of every session. The house light was turned off during all training stages, acquisition, and probe sessions. Briefly, mice underwent a four stepwise training paradigm as follows, (i) Habituation: mice were habituated to the operant chambers to get familiar with the environment for three consecutive days; (ii) Initial touch training (ITT): mice learned to associate stimuli, represented by an illuminated square, presented on the screen with a reward. Each session concluded after 60 min or after the completion of 30 trials; (iii) Must touch training (MTT): they were trained to interact with the screen in order to receive a reward. Each session concluded after 60 min or completion of 30 trials; and (iv) Punish incorrect training (PIT): mice learned to nose-poke the stimulus on the screen for a reward. If a mouse touched a blank response window, the house light turned on and no reward was given. Each session concluded after 30 min or after the completion of 23/30 trials correct (77%) on consecutive days. Once this final pre-training criterion was reached, mice began the LD task training.

##### Training

The stimuli (two white squares) were presented at the intermediate separation. Each mouse was utilized with one side, either left (position 8) or right (position 11), randomly designated as correct and counter-balanced across mice. In the location discrimination reversal (LDR) task, upon completing seven out of eight trials correctly, the correct location side was reversed to the opposite side (Figure 2E). The training session ended after the completion of 60 trials or after 60 min had elapsed. The criterion for advancement was set at the completion of at least 1 reversal in three out of four consecutive sessions (Figure 2E).

##### Probe

Both the high and low separation were used in LD probe sessions (Figure 2F). One side was randomly designated as the correct location side (either left positions 7 and 9 or right positions 10 and 12 based on difficulty separation) and there were no reversals. In each trial, mice were randomly presented with stimuli at either high or low separation positions. The stimuli were not displayed in the same positions for more than three consecutive trials. Like LD training and reversal, touching the stimulus on the correct side resulted in a reward. In each session, mice were given 60 min to complete 40 trials (20 easy and 20 hard distributed randomly throughout the session). Each session had unlimited trials in 60 min but was limited to two reversals (acquisition and one reversal). The numbers of trials to criterion in high and low separation were calculated. The fewer trials to criterion, the better the performance, indicating the pattern separation ability.

### ^1^H Magnetic Resonance Spectroscopy Examination

^1^H magnetic resonance spectroscopy (^1^H-MRS) was used to detect the content of NAA and Cho in medial septal and vertical limb of the diagonal band (MS/VDB) and DG pre and post-treatment by 7T small animal MRI scanner (70/20USR Biospec, Bruker Biospin GMbls, Germany). First, a 3D rapid acquisition with relaxation enhancement (RARE) T2-weighted sequence was performed to determine the basal forebrain and hippocampus location. Based on the image of T2WI, we selected an area in the MS/VDB and DG as the region of interest (ROI) for ^1^H-MRS measurement. The ROI size was set according to the area of MS/VDB and DG as 2.0 mm × 2.0 mm × 2.0 mm and 1.0 mm × 1.0 mm × 1.0 mm. The selected ROI was shimmed, and the water suppression pulse was adjusted for chemical-shift-water suppression (CHESS) prior to the acquisition of point-resolved spectroscopy (PRESS). The parameters of PRESS-^1^H were as follows: repetition time = 1,500 ms, echo time = 20 ms, number of averages = 256, Field of view = 20 mm × 20 mm, slice thickness = 5 mm, slices = 35, and image size = 256 × 256. The spectral data were processed using the software package TOPSPIN (v3.1, Bruker Biospin, Germany). The areas under the peak for NAA, Cho, and creatine (Cr) were calculated automatically using a quantum estimation (QUEST) method with a subtraction approach for background modeling. Using Cr as the criterion (Moon et al., 2016), NAA/Cr and Cho/Cr were statistically evaluated. The position of the metabolites as following: NAA 2.02 parts per million (ppm), Cho 3.2 ppm, and Cr 3.05 ppm.

### Virus and CNO Injections

All viruses were obtained from BrainVTA Co., Ltd. (Wuhan, China), and virus injection was performed as previously described (Zhan et al., 2019). Mice in the 5 × FAD + hM4Di group, 5 × FAD + EA + hM4Di group, and 5 × FAD + EA + Con group were injected with ChAT-EGFP virus (rAAV2/1-chat-EGFP, serotype 1, 1 × 10^13^ particles/ml) in the MS/VDB of the basal forebrain (from Bregma AP:0.9 mm, ML: 0 mm, DV: −4.9 mm). Mice in the 5 × FAD + hM4Di group and 5 × FAD + EA + hM4Di group were injected with the hM4Di DREADD virus [AAV2-Ef1α-DIO-hM4D(Gi)-mCherry, retrotype, 2 × 10^12^ particles/ml] in the DG of the bilateral hippocampus (from Bregma AP: −2.00 mm, ML: ± 1.3 mm, DV: −2.3 mm). Mice in the 5 × FAD + EA + con group were injected with the control virus (AAV2-Ef1α-DIO-mCherry, retrotype, 2 × 10^12^ particles/ml). The virus was delivered using a syringe pump at a rate of 20 nl/min for 10 min, for a total of 200 nl/infusion. The syringe was then raised.2 mm, and remained in place for 15 min after each injection to allow for virus diffusion, and was then slowly retracted. Mice recovered 3 days before behavioral tests.

For activating hM4Di viruses, the stock solution of CNO (BrainVTA Co., Ltd., Wuhan, China) was prepared by dissolving CNO in 100% dimethyl sulfoxide (DMSO) at a concentration of.33 mg/ml and storing at −20°C. On the day of injection, CNO stock was thawed and diluted to.2 ml/20g in a sterile 0.9% saline solution. Mice received intraperitoneal injections of CNO every other day for three times/week during treatment or 30 min prior to MRS examination and behavioral tests after treatment.

Mice were deeply anesthetized with pentobarbital sodium and perfused intracardially with saline, followed by 4% paraformaldehyde (PFA, 16005, Sigma-Aldrich). Brains were removed and post-fixed overnight for 24 h. Free-floating sections (∼30 μm thick, one-fourth of the total sections that contained the MS/VDB and DG) were sliced to observe the cholinergic neural circuit projection and expression of hM4Di/Con viruses by a laser scanning confocal microscope (LSM 710, Carl Zeiss, Germany).

### Nissl Staining

After behavioral tests, mice were sacrificed and brains were removed. Following routine processing in paraffin, coronal sections of the brain were cut at 5 μm thickness in a microtome (Leica, Germany). Nissl staining was performed as previously described (Zhang et al., 2019) to observe neurons morphological in MS/VDB and DG. Results were examined under a microscope (DMI8, Leica, Germany).

### Thioflavin S Staining

Thioflavin S (ThS) staining was performed with paraffin-embedded slices as previously described (Gao et al., 2019) to measure the Aβ plaque. Coronal sections of paraffin-embedded were cut at 5 μm thickness in ThS staining, and results were examined by a confocal. The Aβ plaque area of MS/VDB and DG were calculated by Image J and results are expressed as the area fraction (%) with a formula (plaque area/image area) × 100%.

### Immunohistochemistry

Immunohistochemistry was carried out with paraffin-embedded slices and streptavidin peroxidase kit (SP-0024, Bioss, China) to measure the cholinergic pathway biomarkers, including choline acetyltransferase (ChAT), acetylcholinesterase (AChE), and vesicular acetylcholine transporters (VAChTs). Primary antibodies were as follows: anti-ChAT (1:200, ab6168, Abcam) or anti-AChE (1:50, ab183591, Abcam) or anti-VAChT (1:500, Sab4200559, Sigma) for overnight in 4°C. Slices were dehydrated in 70, 95, and 100% ethanol, cleared in xylene, then covered with neutral resin. Sections of each mouse were taken to acquire images in MS/VDB and DG at high power magnification (200×). Images were analyzed by Image J and results are expressed as mean optical density (MOD). For immunohistochemical detection, negative controls were processed in every immunohistochemistry run.

### Immunofluorescence

Immunofluorescence was performed as protocol (Im et al., 2019). Coronal paraffin-embedded slices were blocked and incubated with primary antibodies as follows: anti-6E10 (1:1,000, 803001, Biolegend), anti-CHRM1 (1:100, BA 1543, Boster), anti-CHRM2 (1:100, ab 109226, Abcam), anti-Doublecortin (DCX, 1:500, ab135349, Abcam), and anti-Neuro-D1 (1:200, ab60704, Abcam) overnight in 4°C. Anti-mouse 488, anti-mouse 555, and anti-rabbit 555 (1:1,000, Thermo Fisher Scientific) were used and nuclei were visualized with DAPI. Sections of each mouse were taken to acquire images in MS/VDB and DG at high power magnification (200×) by confocal microscope. Images were analyzed by Image J and results are expressed as positive areas or cells. Negative controls were processed in every immunofluorescence run.

### Statistical Analysis

All data in the text and Figure legends are represented as the mean ± SEM. All statistical results were analyzed by SPSS 21.0. One-way ANOVA with Dunnett’s multiple comparisons *post hoc* test was used to analyze the effects of EA and hM4Di virus treatment. Paired *t*-test was used to analyze the data pre and post-treatment. Repeated measurement ANOVA was used to analyze repeated measurement data. *P* < 0.05 was considered statistically significant.

## Results

### Electro-Acupuncture Improves Spatial Recognition Memory and Pattern Separation Impairment

**FIGURE 3:**
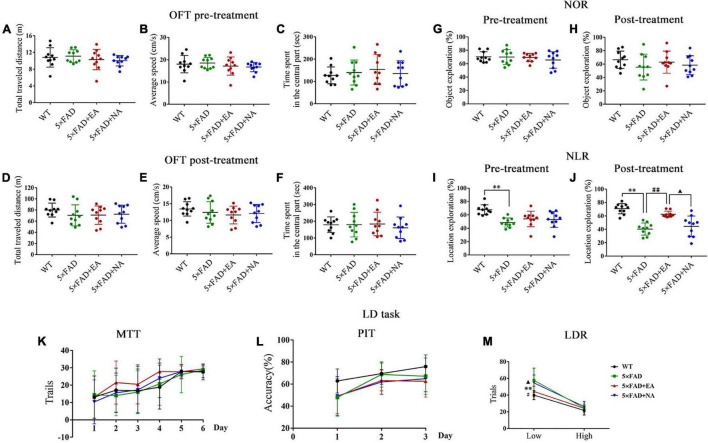
Electro-acupuncture (EA) treatment improves spatial recognition memory and pattern separation impairment in 5 × FAD mice. Results of the open field test (OFT) are expressed as a total traveled distance **(A,D),** average speed **(B,E)** and time spent in the central part **(C,F)** pre and post-treatment (*n* = 10/group). Exploration of mice during novel object recognition (NOR) **(G,H)** and novel location recognition (NLR) **(I,J)** learning paradigms (*n* = 10/group). Results of LD task are expressed as trails of must touch training (MTT) **(K)**, accuracy of punish incorrect training (PIT) **(L)** and trails of location discrimination reversal (LDR) **(M)** (*n* = 8/group). Data are represented as mean ± SD. Each circle represents a mouse (**p < 0.01 vs WT group; ^#^*p* < 0.05 and ^##^*p* < 0.01 vs 5 × FAD group; ^▲^*p* < 0.05 vs 5 × FAD + EA group).

To determine the cognitive impairment in the early stage of AD, we performed a battery of behavioral tests assaying locomotor activity and exploratory behavior in OFT, object recognition memory and spatial recognition memory in NOR and NLR, and pattern separation ability in touchscreen LD task. Total traveled distance, average speed, and time spent in the central part in OFT and object exploration in NOR were not different between groups pre and post-treatment (*p* > 0.050) (Figures 3A–H). In NLR, 5 × FAD group showed a lower location exploration compared with the WT group pre-treatment (*p* < 0.001), and 5 × FAD + EA mice performed significantly better than 5 × FAD mice (*p* < 0.001) and 5 × FAD + NA (*p* = 0.027) mice post-treatment (Figures 3I,J). In the LD task, there was a significant effect due to training days for both the trials in MTT (*p* = 0.819, Figure 3K) and accuracy in PIT (*p* = 0.862, Figure 3L). In LDR, all groups shown similar trials at high separation (*p* = 0.386), whereas the 5 × FAD group had more trials than the WT group (*p* < 0.001), and a significant decrease in trials to reversal of 5 × FAD + EA group in comparison to 5 × FAD group (*p* = 0.033) and 5 × FAD + NA group (*p* = 0.023) at low separation (Figure 3M). These data indicated that EA treatment improved spatial recognition memory and pattern separation impairment in the early stage of 5 × FAD mice.

### Electro-Acupuncture Regulates the Cholinergic System in MS/VDB and DG

**FIGURE 4:**
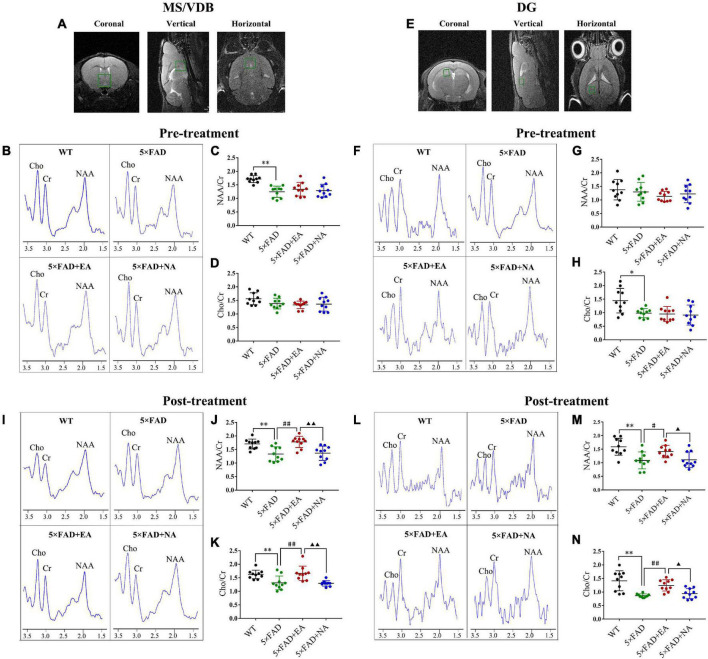
Electro-acupuncture (EA) treatment regulates brain metabolites in medial septal and vertical limb of the diagonal band (MS/VDB) and dentate gyrus (DG) by ^1^H MRS. **(A,E)** The location of the MS/VDB and DG on the T2-weighted scan is the ^1^H MRS shimming region. **(B,F,I,L)** ^1^H MRS exhibits N-acetylaspartate (NAA) peak at 2.02 ppm, choline (Cho) peak at 3.2 ppm, and creatine (Cr) peak at 3.05 ppm in MS/VDB and DG of groups pre and post-treatment. The ratio of NAA/Cr in MS/VDB **(C,D)** and Cho/Cr in DG **(G,H)** were significantly decreased pre-treatment, and the ratios of NAA/Cr and Cho/Cr in MS/VDB **(J,K)** and DG **(M,N)** were significantly increased in the 5 × FAD + EA group. Data are represented as mean ± SD, *n* = 10/group, and each circle represents a mouse (*p < 0.05 and **p < 0.01 vs WT group; ^#^*p* < 0.05 and ^##^*p* < 0.01 vs 5 × FAD group; ▲*p* < 0.05 and ▲▲*p* < 0.01 vs 5 × FAD + EA group).

To detect the cholinergic function, we examined the content of NAA and Cho in MS/VDB and DG as the ROI by ^1^H-MRS (Figures 4A,E). The representative pictures of all groups pre and post-treatment were shown in Figures 4B,F,I,L. Compared with the WT mice, the NAA/Cr in MS/VDB and Cho/Cr in DG were decreased in 5 × FAD group pre-treatment (NAA/Cr, *p* < 0.001; Cho/Cr, *p* = 0.040, Figures 4C,D,G,H). On the other hand, the NAA/Cr and Cho/Cr in MS/VDB and DG were increased in 5 × FAD + EA group compared with the 5 × FAD group (NAA/Cr in MS/VDB, *p* < 0.001; Cho/Cr in MS/VDB, *p* = 0.001; NAA/Cr in DG, *p* = 0.016; Cho/Cr in DG, *p* = 0.001) and 5 × FAD + NA group (NAA/Cr in MS/VDB, *p* < 0.001; Cho/Cr in MS/VDB, *p* < 0.001; NAA/Cr in DG, *p* = 0.023; Cho/Cr in DG, *p* = 0.018) post-treatment (Figures 4J,K,M,N).

**FIGURE 5:**
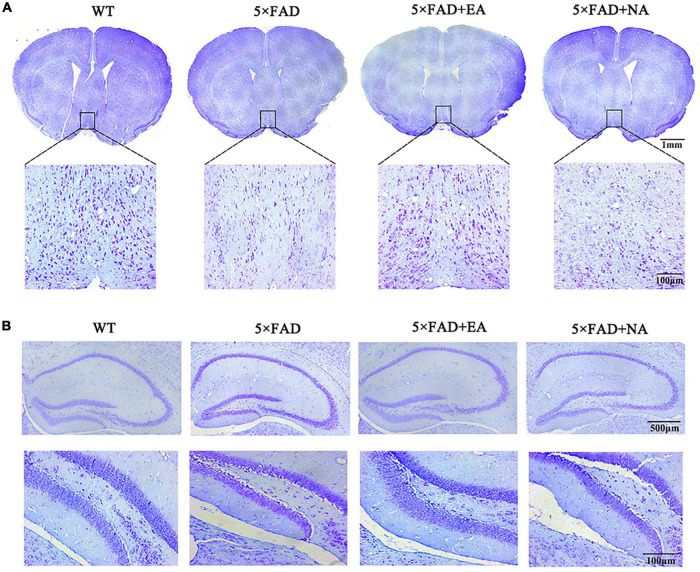
Nissl staining results of WT, 5 × FAD, 5 × FAD + EA, and 5 × FAD + NA group after treatment. **(A)** Nissl staining pictures of basal forebrain level (Top, scale bar = 1 mm) and the black frame area indicates the field of view of the MS/VDB. 200× photomicrographs of MS/VDB (Bottom, scale bar = 100 mm). **(B)** Nissl staining pictures in the hippocampus (Top, scale bar = 50 μm) and 200× photomicrographs of DG (Bottom, scale bar = 100 μm).

We next observed the morphological integrity of nerve cells by Nissl staining. The neurons of the WT group and 5 × FAD + EA group in the MS/VDB were arranged more neatly, with darker and more obvious Nissl bodies. Moreover, the 5 × FAD group and 5 × FAD + NA group neurons in the MS/VDB were sparsely arranged with light Nissl body staining (Figure 5A). In contrast, the neurons of groups in the DG were arranged orderly, with complete morphology and uniform staining (Figure 5B).

**FIGURE 6:**
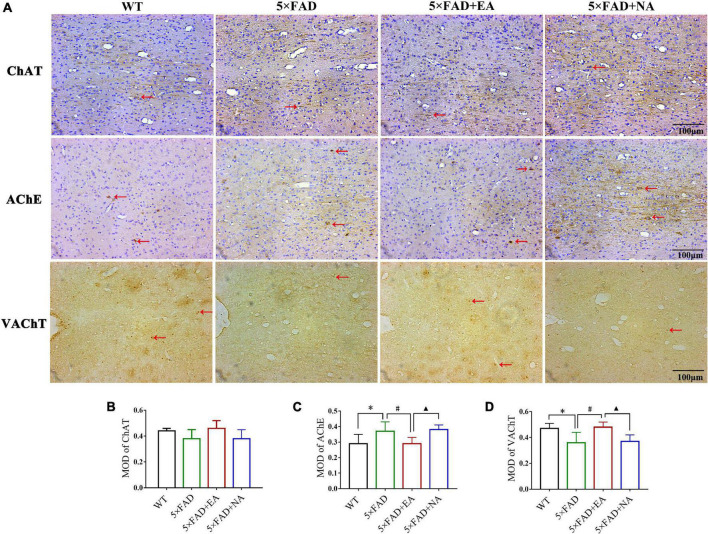
Electro-acupuncture (EA) treatment regulates cholinergic biomarkers of MS/VDB by immunohistochemistry. ChAT and AChE express in the cytoplasm and nerve fibers (brown), and the nuclei are stained with hematoxylin (blue). VAChT expression is granular (brown), without hematoxylin staining of the nuclei. **(A)** Representative 200× photomicrographs of choline acetyltransferase (ChAT) (Top), enzyme acetylcholinesterase (AChE) (Middle), and vesicular acetylcholine transporter (VAChT) (Bottom) expression. **(B–D)** Statistical results of mean optical density (MOD) of ChAT, AChE and VAChT in MS/VDB. Red arrows indicate positive protein expression, scale bar = 100 μm. Data are represented as mean ± SD, *n* = 4/group (*p < 0.05 and **p < 0.01 vs WT group; #*p* < 0.05 vs 5 × FAD group; ▲*p* < 0.05 vs 5 × FAD + EA group). ChAT, choline acetyltransferase; AChE, enzyme acetylcholinesterase; VAChT, vesicular acetylcholine transporter; MOD, mean optical density.

**FIGURE 7:**
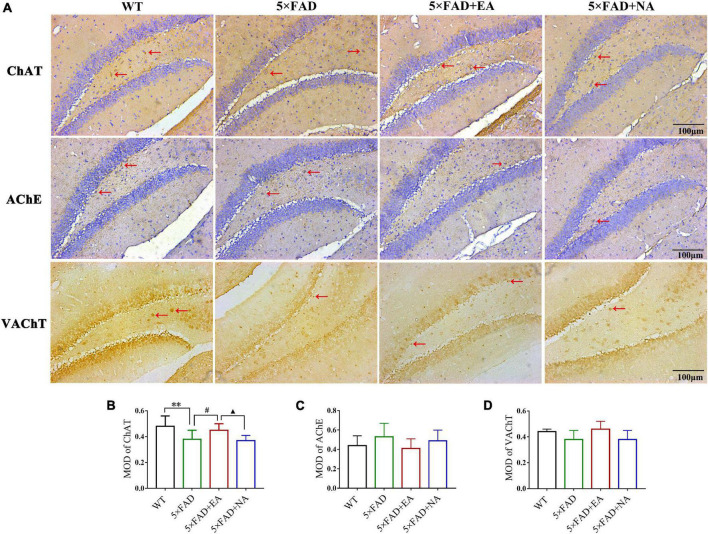
Electro-acupuncture (EA) treatment regulates cholinergic biomarkers of DG by immunohistochemistry. ChAT and AChE express in the cytoplasm and nerve fibers (brown), and the nuclei are stained with hematoxylin (blue). VAChT expression is granular (brown), without hematoxylin staining of the nuclei. **(A)** Representative 200 × photomicrographs of ChAT (Top), AChE (Middle), and VAChT (Bottom) expression. **(B–D)** Statistical results of MOD of ChAT, AChE and VAChT in DG. Red arrows indicate positive protein expression, scale bar = 100 μm. Data are represented as mean ± SD, *n* = 4/group (*p < 0.05 and **p < 0.01 vs WT group; ^#^*p* < 0.05 vs 5 × FAD group; ▲*p* < 0.05 vs 5 × FAD + EA group). ChAT, choline acetyltransferase; AChE, enzyme acetylcholinesterase; VAChT, vesicular acetylcholine transporter; MOD, mean optical density.

We subsequently examined the expression and distribution of cholinergic biomarkers by Immunohistochemistry. The immunopositive reaction was observed as deep brown staining and MOD was measured in MD/VDB (Figure 6) and DG (Figure 7). ChAT and AChE were expressed in the cytoplasm and nerve fibers, and VAChT expression was granular. In MS/VDB, MOD of ChAT was no significant difference between groups (*p* = 0.141). Compared with the WT group, MOD of AChE was increased (*p* = 0.042) but MOD of VAChT was decreased in the 5 × FAD group (*p* = 0.014). Mice in the 5 × FAD + EA group has less AChE expression compared with the 5 × FAD group (*p* = 0.030) and 5 × FAD + NA group (*p* = 0.022) but had more VAChT expression (*p* < 0.050). In DG, MOD of AChE and VAChT had no significant difference between groups (AChE, *p* = 0.218; VAChT, *p* = 0.618). MOD of ChAT was decreased in 5 × FAD group compared with the WT group (*p* = 0.003). Mice in the 5 × FAD + EA group had more ChAT expression than the 5 × FAD group (*p* = 0.030) and 5 × FAD + NA group (*p* = 0.018).

These data indicated that EA treatment regulated the function of the cholinergic system in the early stage of 5 × FAD mice.

### Electro-Acupuncture Decreases Aβ Plaques Deposition in DG

**FIGURE 8:**
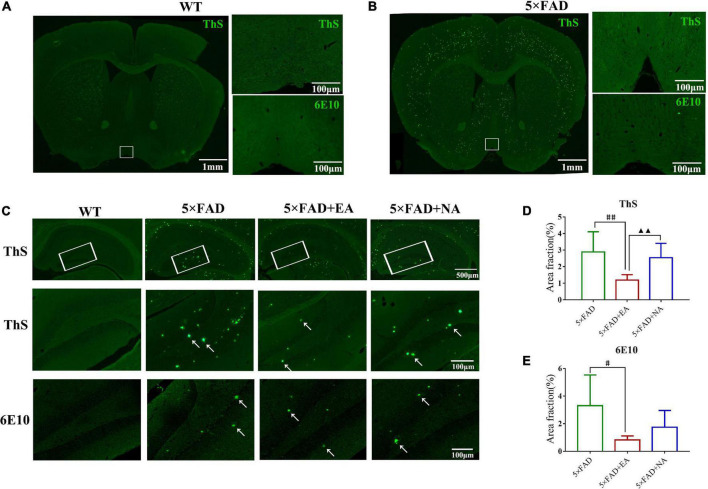
Electro-acupuncture (EA) treatment modulates Aβ deposition in 5 × FAD mice. **(A,B)** Representative images of Aβ plaques in the basal forebrain (Left) and the white frame area indicate the view of the MS/VDB of WT mice and 5 × FAD mice (scale bar = 1mm). 200× photomicrographs of Thioflavin S (ThS) and anti-Aβ (6E10) immunostaining in the MS/VDB were shown (Right, scale bar = 100 μm). **(C)** Representative images of Aβ plaques in the hippocampus and the white frame area indicate the view of the DG (Top) (scale bar = 500 μm). 200× photomicrographs of ThS (Middle), and anti-Aβ (6E10) (Bottom) immunostaining in DG were shown (scale bar = 100 μm). White arrows indicate Aβ plaques. **(D,E)** Quantitative analysis of area fraction by ThS and anti-Aβ (6E10). Data are represented as mean ± SD, *n* = 4/group (^#^*p* < 0.05 and ^##^*p* < 0.01 vs 5 × FAD group; ▲▲*p* < 0.01 vs 5 × FAD + EA group). ThS: Thioflavin S.

We evaluated whether EA treatment could eliminate Aβ pathology by ThS and 6E10 immunofluorescence. First, we observed Aβ plaques expression of WT group and 5 × FAD group in MS/VDB. The result of the whole-brain picture shown that there was no Aβ deposition of wild type mice (by ThS, Figure 8A), whereas a large number of Aβ plaques of 5 × FAD mice were observed and scattered in the cerebral cortex, lateral septal nucleus, and other brain areas (by ThS, Figure 8B). A further observation under high magnification found no evident Aβ plaque expression in MS/VDB, and no fluorescence occurred. Next, we observed the Aβ pathology of whole-hippocampus. Images by ThS showed that Aβ deposition appeared in the 5 × FAD group, 5 × FAD + EA group, and 5 × FAD + NA group (Figure 8C). We observed Aβ plaques and counted the Aβ fraction ratio of DG at high magnification. A significant Aβ fraction ratio reduction of 5 × FAD + EA group compared with the 5 × FAD group (ThS, *p* = 0.001; 6E10, *p* = 0.035) was observed. Furthermore, there was a lower Aβ fraction ratio in the 5 × FAD + EA group than those in the 5 × FAD + NA group (ThS, *p* = 0.001, Figure 8D; 6E10, *p* = 0.179, Figure 8E). These data indicated that EA treatment decreased the Aβ fraction ratio in DG rather than MS/VDB in the early stage of 5 × FAD mice.

### The Expression of MS/VDB-DG Cholinergic Neural Circuit and Chemogenetic Virus Expression

**FIGURE 9:**
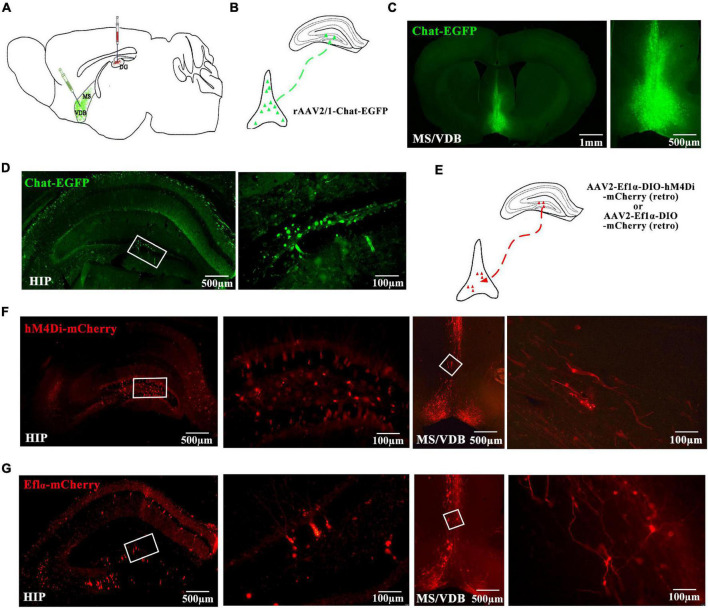
The MS/VDB-DG cholinergic neural circuit projections and hM4Di receptor expression. **(A)** Schematic of the virus injections in the MS/VDB and DG. **(B)** Schematic of the monosynaptic anterograde tracing strategy shows the rAAV2/1-chat-EGFP virus in MS/VDB for labeling ChAT neurons. **(C,D)** Representative images of the basal forebrain (Left) (scale bar = 1 mm) and hippocampus (Left) (scale bar = 500 μm). photomicrographs of MS/VDB (Right) (scale bar = 500 μm) and DG (Right) (scale bar = 100 μm). **(E)** Schematic of the monosynaptic retrograde tracing strategy shows the application of AAV2-Ef1α-DIO-hM4D(Gi)-mCherry or AAV2-Ef1α-DIO-mCherry virus in DG. **(F,G)** Chemogenetic inhibition of the MS/VDB-DG cholinergic neural circuit and representative image of the hM4Di-mCherry expression in the DG (Left) and MS/VDB (Right). Ef1α-mCherry set as control. The white frame indicates 200× photomicrographs.

Next, we focused on the neural mechanism of EA treatment via inhibiting the MS/VDB-DG cholinergic neural circuit in the early AD stage. We injected a monosynaptic anterograde adeno-associated virus (AAV) in MS/VDB (Figures 9A,B), which encodes enhanced green fluorescence protein (EGFP) and expresses in ChAT neurons. After behavioral tests and MRS scan, mice were sacrificed and brain sections were processed. A bright green signal (EGFP) was detected in MS/VDB and DG of mice (Figures 9C,D).

A monosynaptic retrograde AAV virus-encoded hM4D(Gi) and/or mCherry was injected into the DG of the bilateral hippocampus (Figures 9A,E). hM4Di DREADDs are mutated muscarinic acetylcholine receptors that act through G-protein coupling receptor signaling cascades. In our study, inhibitory G-protein coupling receptor signaling cascades were acted to affect neuronal activity upon activation by CNO. The brain sections were processed and a red signal (mCherry) was detected in DG and MS/VDB (Figures 9F,G).

### Electro-Acupuncture Activates the MS/VDB-DG Cholinergic Neural Circuit Improving Pattern Separation Impairment

**FIGURE 10:**
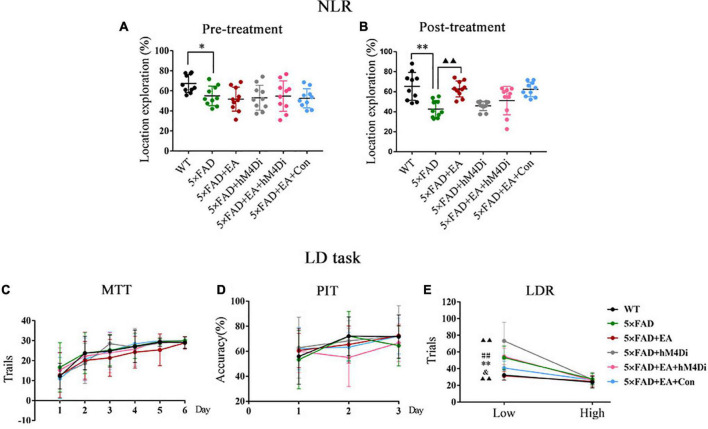
Electro-acupuncture (EA) treatment activates the MS/VDB-DG cholinergic neural circuit improving the pattern separation impairment in 5 × FAD mice. Exploration of mice during novel location recognition (NLR) pre-treatment **(A)** and post-treatment **(B)**. Each circle represents a mouse, and *n* = 10/group. Results of the location discrimination (LD) task are expressed as trails of MIT **(C)**, the accuracy of PIT **(D)**, and trails of LDR **(E)** (*n* = 8/group). Data are represented as mean ± SD (*p < 0.05 and ^**^p < 0.01 vs WT group; ▲▲*p* < 0.01 vs 5 × FAD group; ^##^*p* < 0.01 vs 5 × FAD + EA group; ^&^*p* < 0.05 vs 5 × FAD + EA + hM4Di group). NLR, novel location recognition; MTT, must touch training; PIT, punish incorrect training; LDR, location discrimination reversal.

The effects of long-term CNO-mediated hM4Di DREADDs inhibition of the MS/VDB-DG cholinergic neural circuit on the cognitive-behavioral after EA treatment were investigated. In NLR, the 5 × FAD group shown a lower location exploration than the WT group pre-treatment (*p* = 0.020), and 5 × FAD + EA mice performed significantly better than 5 × FAD mice post-treatment (*p* = 0.001, Figures 10A,B). The location exploration of mice between the 5 × FAD + EA group and 5 × FAD + EA + hM4Di group (*p* = 0.385), the 5 × FAD group and 5 × FAD + hM4Di group (*p* = 0.989), and the5 × FAD + EA + hM4Di group and 5 × FAD + EA + Con group (*p* = 0.414) had no significant difference.

Moreover, in the LD task, the trials in MTT (Figure 10C) and accuracy in PIT (Figure 10D) were increasing with training days. There was no significant difference between groups (MTT, *p* = 0.888; PIT, *p* = 0.358). In LDR, mice in all groups showed similar trials at high separation (*p* = 0.896), whereas the 5 × FAD group had more trials than the WT group (*p* = 0.002). After EA treatment, mice in the 5 × FAD + EA group had fewer trials than 5 × FAD group (*p* = 0.001). There was a significant increase in the trials to reversal of 5 × FAD + EA + hM4Di group in comparison to 5 × FAD + EA group (*p* = 0.001) and 5 × FAD + EA + Con group (*p* = 0.032) at low separation (Figure 10E).

These data indicated that EA treatment activated the MS/VDB-DG cholinergic neural circuit to improve pattern separation impairment rather than spatial recognition memory deficit in the early stage of 5 × FAD mice.

### Electro-Acupuncture Activates the MS/VDB-DG Cholinergic Neural Circuit Regulating the Cholinergic System

**FIGURE 11:**
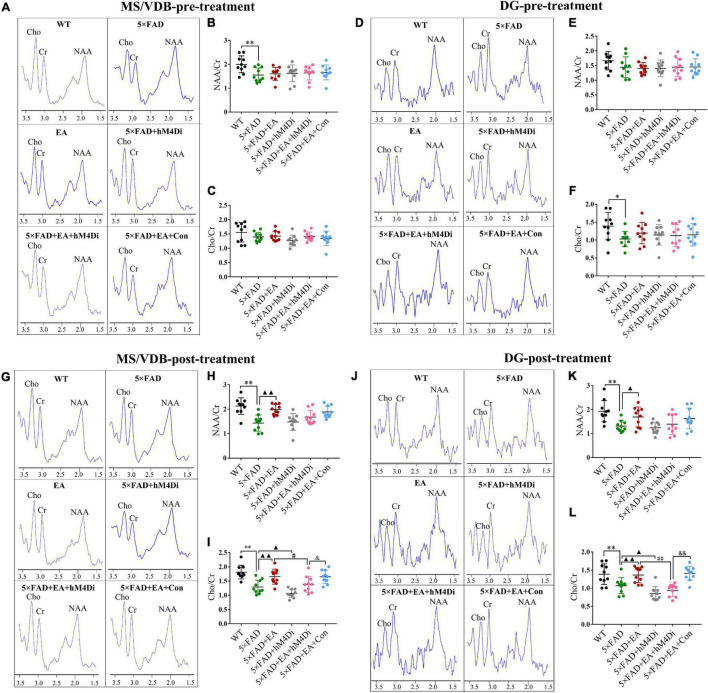
Electro-acupuncture (EA) treatment activates the MS/VDB-DG regulating Cho/Cr in 5 × FAD mice. **(A,D,G,J)** ^1^H MRS exhibits NAA peak at 2.02 ppm, Cho peak at 3.2 ppm, and Cr peak at 3.05 ppm in MS/VDB and DG of groups pre- and post-treatment. The ratio of NAA/Cr in MS/VDB **(B,C)** and Cho/Cr in DG **(E,F)** were significantly decreased pre-treatment. The ratios of NAA/Cr and Cho/Cr in MS/VDB **(H,I)** and DG **(K,L)** were significantly increased in the 5 × FAD + EA group. With inhibition of the MS/VDB-DG cholinergic neural circuit, the ratios of Cho/Cr in MS/VDB and DG were decreased. Data are represented as mean ± SD, n = 10/group, and each circle represents a mouse (*p < 0.05 and ^**^p < 0.01 vs WT group; ▲*p* < 0.05 and ▲▲*p* < 0.01 vs 5 × FAD group; ^#^*p* < 0.05 and ^##^*p* < 0.01 vs 5 × FAD + EA group; ^&^*P* < 0.05 and ^&&^*p* < 0.01 vs 5 × FAD + EA + hM4Di group).

Magnetic resonance spectrometry (MRS) and muscarinic cholinergic receptors were examined to assess the effects of long-term hM4Di DREADDs inhibition of the MS/VDB-DG cholinergic neural circuit after EA treatment. The representative pictures of MRS in MS/VDB and DG pre and post-treatment were shown in Figures 11A,D,G,J. Since the cycle of AAV virus transfection at least 28 days, there is no significant difference of NAA/Cr and Cho/Cr between groups except for the WT group pre-treatment (*p* > 0.050, Figures 11B,C,E,F). With the inhibition of the MS/VDB-DG cholinergic neural circuit, Cho/Cr in MS/VDB and DG were decreased in 5 × FAD + hM4Di group (MS/VDB, *p* = 0.037; DG, *p* = 0.031) and 5 × FAD + EA + hM4Di group (MS/VDB, *p* = 0.014; DG, *p* < 0.001, Figures 11I,L). Compared with the 5 × FAD + EA + hM4Di group mice, the Cho/Cr was increased in the 5 × FAD + EA + Con group (MS/VDB, *p* = 0.015; DG, *p* < 0.001). Besides, we found no significant difference of NAA/Cr by inhibiting the MS/VDB-DG cholinergic neural circuit post-treatment (*p* > 0.050, Figures 11H,K).

**FIGURE 12:**
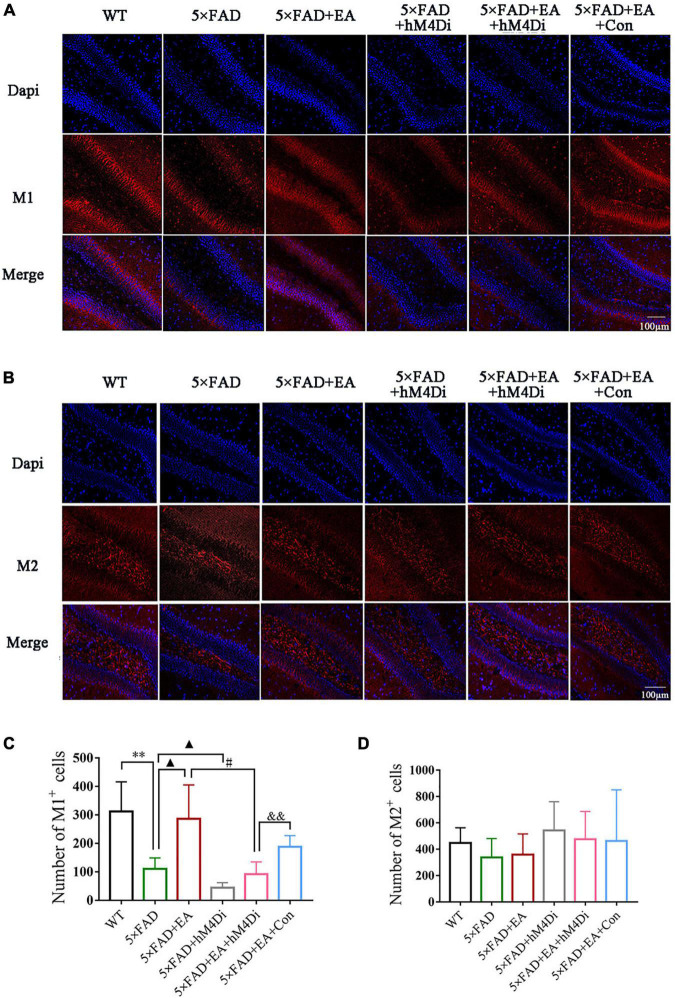
Electro-acupuncture (EA) treatment activates the MS/VDB-DG cholinergic neural circuit regulating M1 receptors in 5 × FAD mice. Representative images of M1 receptors **(A)** and M2 receptors **(B)** in the DG. Groups were subjected to immunofluorescence labeling with Dapi (Blue), M1 or M2 (red) staining. Scale bar = 100 μm. Quantified M1 positive cells **(C)** and M2 positive cells **(D)**. Data are represented as mean ± SD, *n* = 4/group (^**^p < 0.01 vs WT group; ▲*p* < 0.05 vs 5 × FAD group; ^#^*p* < 0.05 vs 5 × FAD + EA group; ^&&^*p* < 0.01 vs 5 × FAD + EA + hM4Di group).

Next, we investigated muscarinic cholinergic receptors (M1 and M2) in the DG of hippocampus of the mice by immunofluorescence (Figures 12A,B). Morphometric analysis revealed a significantly lower number of M1^+^ cells in 5 × FAD group mice than WT mice (*p* = 0.008), and the number of M1^+^ cells increased after EA treatment (*p* = 0.040, Figure 12C). With the inhibition of the MS/VDB-DG cholinergic neural circuit, the M1 positive cells in the 5 × FAD + EA + hM4Di group were significantly decreased compared with the 5 × FAD + EA group (*p* = 0.023). M1 positive cells of the 5 × FAD + EA + hM4Di group was lower than those in the 5 × FAD + EA + Con group (*p* = 0.005). Furthermore, there was no significant difference of M2 positive cells in either EA treatment or inhibition of the MS/VDB-DG cholinergic neural circuit between groups (*p* = 0.396, Figure 12D).

These data indicated that EA treatment activated the MS/VDB-DG neural circuit to regulate the function of the cholinergic system in the early stage of 5 × FAD mice.

### Electro-Acupuncture Activates the MS/VDB-DG Cholinergic Neural Circuit Promoting Hippocampus Neurogenesis

**FIGURE 13:**
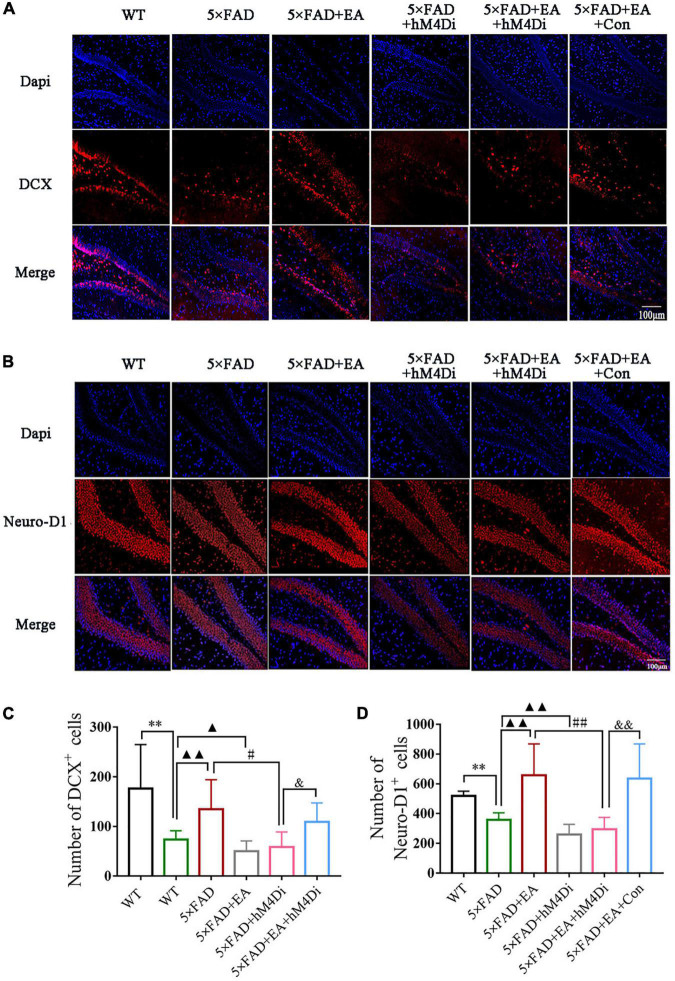
EA treatment activates the MS/VDB-DG cholinergic neural circuit promoting hippocampus neurogenesis in 5 × FAD mice. Representative images of doublecortin (DCX) **(A)** and Neuro-D1 **(B)** in the DG. Groups were subjected to immunofluorescence labeling with Dapi (Blue), DCX or Neuro-D1 (red) staining. Scale bar = 100 μm. Quantified DCX positive cells **(C)** and Neuro-D1 positive cells **(D)**. Data are represented as mean ± SD, *n* = 4/group (^**^p < 0.01 vs WT group; ▲*p* < 0.05 and ▲▲*p* < 0.01 vs 5 × FAD group; ^#^*p* < 0.05 and ^##^*p* < 0.01 vs 5 × FAD + EA group; ^&^*p* < 0.05 and ^&&^*p* < 0.01 vs 5 × FAD + EA + hM4Di group). DCX, doublecortin; Neuro-D1, neurogenic differentiation factor 1.

Anti-DCX and anti-Neuro-D1 antibody were used by immunofluorescence to confirm neurogenesis in the hippocampus (Figures 13A,B). Compared with the WT group, the number of DCX^+^ cells and Neuro-D1^+^ cells were decreased in the 5 × FAD group (DCX, *p* < 0.001, Figure 13C; Neuro-D1, *p* < 0.001; Figure 13D). After EA treatment, DCX^+^ cells and Neuro-D1^+^ cells were increased in the 5 × FAD + EA group (DCX, *p* = 0.003; Neuro-D1, *p* = 0.001). With the inhibition of the MS/VDB-DG cholinergic neural circuit, the number of DCX^+^ cells and Neuro-D1^+^ cells in the 5 × FAD + hM4Di group and 5 × FAD + EA + hM4Di group were significantly decreased compared with the 5 × FAD group and 5 × FAD + EA group (DCX, *p* < 0.050; Neuro-D1, *p* < 0.001). Furthermore, the number of DCX^+^ cells and Neuro-D1^+^ cells of the 5 × FAD + EA + hM4Di group declined compared with the 5 × FAD + EA + Con group (DCX, *p* = 0.049; Neuro-D1, *p* < 0.001).

These data indicated that EA treatment activated the MS/VDB-DG cholinergic neural circuit to promote hippocampus neurogenesis in the early stage of 5 × FAD mice.

## Discussion

**FIGURE 14:**
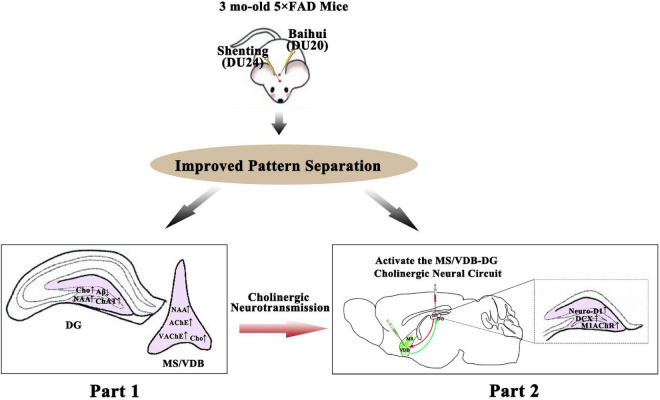
Summary drawing of the experimental findings. The present study included two parts: Baihui (DU20) and Shenting (DU24) acupoints with EA stimulation in 3-month-old 5 × FAD mice **(upper)**, and the pattern separation ability was improved after EA treatment **(middle)**. It was found that the alteration of the cholinergic system in MS/VDB and DG **(left, bottom)**, the increased of the M1 muscarinic receptors and hippocampal neurogenesis in the DG via activating of the MS/VDB-DG cholinergic neural circuit **(right, bottom)**. EA treatment normalized the pattern separation performance in FAD mice, which was related to activating the MS/VDB-DG neural circuit, regulating the M1 muscarinic receptors and promoting hippocampal neurogenesis in DG.

The present study explored the cholinergic neural circuit mechanism underlying pattern separation improvement by EA at DU20 and DU24 acupoints in 5 × FAD mice with the following key findings. First, we demonstrated that EA at DU20 and DU24 acupoints could improve spatial recognition memory and pattern separation impairment accompanied by cholinergic function amelioration and Aβ pathology reduction in the early stage of AD. Next, we proved that the inhibition of the MS/VDB-DG cholinergic neural circuit was able to counteract effects of EA treatment on the pattern separation improvement, M1 receptors increase, and hippocampus neurogenesis. EA, probably by activating the MS/VDB-DG cholinergic neural circuit to regulate the M1AChR-mediated cholinergic neural signal and promote hippocampal neurogenesis in the DG, normalized the pattern separation in the early stage of AD. A schematic summary of the overall experimental findings is presented in Figure 14.

The 5 × FAD mouse model is an early-onset transgenic model that rapidly recapitulates AD amyloid pathology and hippocampal-dependent memory deficits. The present study assessed the locomotor activity and exploratory ability, object recognition memory, spatial recognition memory, and pattern separation of 5 × FAD mice. Behavioral tests indicate that hippocampus-dependent spatial recognition memory, but not object recognition memory, is impaired in 5 × FAD mice in the early ages, which is consistent with previous studies (Rekik et al., 2017). Furthermore, in 5 × FAD mice treated with EA treatment, the exploration of location was increased and the number of trials to reversal was decreased. Although the previous study had proved that EA treatment improves spatial recognition memory (Fan et al., 2015), this study showed that EA treatment might be a novel approach for ameliorating pattern separation impairment in AD.

According to the cholinergic hypothesis, the deterioration of cholinergic neurons and the loss of neurotransmission are the major causes of the decline in cognitive function in the early stage of patients with AD. Patients with AD usually suffer the loss of cholinergic neurons in the hippocampus and cortex (Schliebs and Arendt, 2011) and hence, the decreased cholinergic activity (Khan, 2009), which is possibly due to increased activity AChE. Severely diminished ChAT expression was also observed in the process (Orta-Salazar et al., 2014). Elevation of the ACh levels by pharmacological inhibition of AChE improves behavioral performance in AD (Sharma, 2019). In this study, EA at DU20 and DU24 acupoints could prevent neuron loss in MS/VDB, regulate NAA/Cr and Cho/Cr level, decrease AChE protein expression, and increase ChAT and VAChT in 5 × FAD + EA group mice. Therefore, the cholinergic system was effectively protected by EA treatment, thereby, early cognitive deficits were mitigated.

Cholinergic signaling from the MS/VDB to the hippocampus is certainly important for the formation of spatial memories. Particularly, ACh has consistently been shown by microdialysis to be elevated in the hippocampus during performance of various memory tasks, and numerous studies have probed the effects of exogenous and more recently, endogenous ACh on hippocampal plasticity and performance in spatial memory tasks (Nelson and Mooney, 2016). Zhu reported that choline neurons in the MS/VDB directly innervate newly generated immature neurons in the hippocampus of adult mice and regulate both the survival and spatial pattern impairment of newly generated immature neurons (Zhu et al., 2017). We used a monosynaptic tracing and inhibiting strategies that selectively target cholinergic neurons to illustrate the mechanism of EA treatment that improves cognitive deficits in the early stage of 5 × FAD mice. Via these strategies, the cholinergic neural circuit was inhibited leading to pattern separation impairment in the 5 × FAD + hM4Di group mice. Furthermore, the results of the LD task showed that the cholinergic neural circuit was one of the targets of EA treatment to improve the early pattern separation impairment in 5 × FAD mice. Furthermore, it is shown that stimulation of cholinergic neurons in the MS led both to signaling via a direct cholinergic basal forebrain - hippocampal projection and to the recruitment of an indirect cholinergic to GABAergic basal forebrain to the hippocampal pathway (Dannenberg et al., 2015). Our study focused on the MS/VDB-DG cholinergic circuit and did not observe the role of other receptors in the basal forebrain.

There are increasing reports consistent with cholinergic transmission being mediated by fast synaptic release and consequent activation of postsynaptic AChRs (Nelson and Mooney, 2016). Muscarinic ACh receptors are class receptors of ACh signals, which are G-protein coupled receptors. M1 and M2 are the most abundant muscarinic ACh receptors subtypes in the central nervous system, which are significantly correlated with memory impairments (Zhang et al., 2007). M1 receptors have long been identified as a potential therapeutic target for treating cognitive impairment in AD (Konar et al., 2019; Zhao et al., 2019). M2 receptors play a major role in modulating cholinergic tone, and broadly impacting circuit function and M2 receptors are also the most likely candidates to mediate the muscarinic suppression of GABA release in the hippocampus (Lebois et al., 2018). Therefore, M1 and M2 ACh receptors were selected to observe the effect of EA treatment in the present study. We found that EA treatment increased M1 receptor expression in 5 × FAD mice while inhibiting the MS/VDB-DG cholinergic neural circuit, therefore, M1 receptors expression was reduced. However, whether EA treatment or inhibition of cholinergic neural circuit was done, the level of M2 receptors did not significantly alter in the DG.

M1 and M2 receptors have different effects on the G-proteins signal transduction pathway, leading to different effects of EA treatment on M1 and M2 receptors. M1 classically signal through Gq/11 G-proteins to mediate the excitatory neuromodulatory actions of ACh, while M2 signal through Gi/o G-proteins to mediate the inhibitory neuromodulatory actions of Ach (Jiang et al., 2014). It has been reported that Huangjing and Dilong extract could improve the learning and memory ability in AD rats, which relates to the expression levels of M1 receptors increase, but it does not relate to the expression of M2 receptors (Xiao, 2017). Combined with the results of the present study, it is indicated that M1 receptors, and not M2 receptors, mediate cognitive function improvement.

Hippocampus neurogenesis has been well-documented to play a pivotal role in pattern separation (França et al., 2017). Thus, we performed immunofluorescence staining to evaluate the neurogenesis via DCX and Neuro-D1 labeled. DCX and Neuro-D1 positive cells were markedly mitigated in the 5 × FAD group compared with the WT group but significantly elevated in the 5 × FAD + EA group. While inhibition of cholinergic neural circuit leads to DCX positive cells and Neuro-D1 positive cells reduction in the 5 × FAD + EA + hM4Di group, the above results demonstrated that EA treatment facilitates hippocampus neurogenesis in the early stage of 5 × FAD mice. Moreover, EA treatment has been reported to improve neurogenesis after stroke (Kim et al., 2014). The mechanism of EA treatment which promote neurogenesis remain largely unknown, and the association between EA treatment and cognition warrants further study.

To date, the administration of AChE inhibitors has been used for AD therapy to increase ACh concentration in the brain. Since these drugs are only palliative, other therapeutic approaches are expected to replace the drugs. However, it is believed that the ideal therapeutic strategy for AD should include the recovery of cognitive function and neuroprotection against progressive tissue destruction and neuroregeneration (Kim et al., 2020). Recent studies of ours have demonstrated that the EA treatment improved cognitive impairment in mice with AD (Liu et al., 2017; Lin et al., 2018; Li et al., 2020). In the present study, we demonstrated that the MS/VDB-DG cholinergic neural circuit involved in the EA at DU20 and DU24 acupoints improved the early pattern separation in 5 × FAD mice, which was related to the regulation of the cholinergic system and the promotion neurogenesis. This study provides visual evidence of the effectiveness of EA treatment based on the cholinergic neural circuit to reveal the scientific essence of EA treatment for AD partially. We did not dissect the function of newborn neurons in DG in local networks, but it will be verified in a further study. We continue to find how cholinergic inputs with EA treatment in AD affect newborn neurons in DG.

## Data Availability Statement

The raw data supporting the conclusions of this article will be made available by the authors, without undue reservation.

## Ethics Statement

This study was approved by the Ethical Committee on Animal Experimentation, Fujian University of Traditional Chinese medicine.

## Author Contributions

JT and LC conceived and designed the study. LL performed all experiments and partially wrote the manuscript. JL, YD, and SL discussed the EA treatment and contributed to the data analysis. MY and ZW performed EA experiments. WL designed this study and provided the final manuscript. All authors read and approved the final manuscript.

## Conflict of Interest

The authors declare that the research was conducted in the absence of any commercial or financial relationships that could be construed as a potential conflict of interest.

## Publisher’s Note

All claims expressed in this article are solely those of the authors and do not necessarily represent those of their affiliated organizations, or those of the publisher, the editors and the reviewers. Any product that may be evaluated in this article, or claim that may be made by its manufacturer, is not guaranteed or endorsed by the publisher.

## Abbreviations

5 × FAD: five familial mutation
AAV: adeno-associated virus
A β: amyloid- β
ACh: acetylcholine
AChE: enzyme acetylcholinesterase
AD: Alzheimer’s disease
ChAT: choline acetyltransferase
Cho: choline
Cr: creatine
CNO: clozapine-N-oxide
DCX: doublecortin
DG: dentate gyrus
DU20: baihui
DU24: shenting
EA: electro-acupuncture
^1^H-MRS: PRESS-^1^H Magnetic resonance spectroscopy
ITT: initial touch training
LD: location discrimination
LDR: location discrimination reversal
MS: medial septum
MTT: must touch training
NAA: N-acetylaspartate
Neuro-D1: neurogenic differentiation factor 1
NLR: novel location recognition
NOR: novel object recognition
OFT: open field test
PIT: punish incorrect training
ThS: thioflavin S
VAChT: vesicular acetylcholine transporter
VDB: vertical limb of the diagonal band of Broca.

## References

[B1] Cayco-Gajic N. A. Silver R. A. (2019). Re-evaluating Circuit Mechanisms Underlying Pattern Separation. *Neuron* 101 584–602. 10.1016/j.neuron.2019.01.044 30790539 PMC7028396

[B2] Dannenberg H. Pabst M. Braganza O. Schoch S. Niediek J. Bayraktar M. (2015). Synergy of direct and indirect cholinergic septo-hippocampal pathways coordinates firing in hippocampal networks. *J. Neurosci.* 35 8394–8410. 10.1523/JNEUROSCI.4460-14.2015 26041909 PMC6605336

[B3] Fan X. W. Chen F. Chen Y. Chen G. H. Liu H. H. Guan S. K. (2015). Electroacupuncture prevents cognitive impairments by regulating the early changes after brain irradiation in rats. *PLoS One* 10:e0122087. 10.1371/journal.pone.0122087 25830357 PMC4382177

[B4] Ferreira-Vieira T. H. Guimaraes I. M. Silva F. R. Ribeiro F. M. (2016). Alzheimer’s disease: Targeting the Cholinergic System. *Curr. Neuropharmacol.* 14 101–115. 10.2174/1570159x13666150716165726 26813123 PMC4787279

[B5] França T. Bitencourt A. M. Maximilla N. R. Barros D. M. Monserrat J. M. (2017). Hippocampal neurogenesis and pattern separation: A meta-analysis of behavioral data. *Hippocampus* 27 937–950. 10.1002/hipo.22746 28597491

[B6] Frank D. Montemurro M. A. Montaldi D. (2020). Pattern Separation Underpins Expectation-Modulated Memory. *J. Neurosci.* 40 3455–3464. 10.1523/JNEUROSCI.2047-19.2020 32161140 PMC7178906

[B7] Gao Y. Liu Q. Xu L. Zheng N. He X. Xu F. (2019). Imaging and Spectral Characteristics of Amyloid Plaque Autofluorescence in Brain Slices from the APP/PS1 Mouse Model of Alzheimer’s Disease. *Neurosci. Bull* 35 1126–1137. 10.1007/s12264-019-00393-6 31127445 PMC6864001

[B8] Han Y. G. Qin X. Zhang T. Lei M. Sun F. Y. Sun J. J. (2018). Electroacupuncture prevents cognitive impairment induced by lipopolysaccharide via inhibition of oxidative stress and neuroinflammation. *Neurosci. Lett.* 683 190–195. 10.1016/j.neulet.2018.06.003 29885447

[B9] Huang S. Li L. Wang Z. F. Yang M. G. Li J. H. Zhang J. Y. (2019). Effect of Electroacupuncture on Learning memory and Expression of β-site Amyloid Precursor Protein Cleaving Enzyme 1 in APP/PS1 Mice. *Chin. J. Rehabil. Theory Pract.* 25 44–50. 10.3969/j.issn.1006-9771.2019.01.007

[B10] Im K. Mareninov S. Diaz M. Yong W. H. (2019). An Introduction to Performing Immunofluorescence Staining. *Methods Mol. Biol.* 1897 299–311. 10.1007/978-1-4939-8935-5_26 30539454 PMC6918834

[B11] Jawhar S. Trawicka A. Jenneckens C. Bayer T. A. Wirths O. (2012). Motor deficits, neuron loss, and reduced anxiety coinciding with axonal degeneration and intraneuronal Aβ aggregation in the 5×FAD mouse model of Alzheimer’s disease. *Neurobiol. Aging* 33 .e29–.e40. 10.1016/j.neurobiolaging.2010.05.027 20619937

[B12] Jiang S. Li Y. Zhang C. Zhao Y. Bu G. Xu H. (2014). M1 muscarinic acetylcholine receptor in Alzheimer’s disease. *Neurosci. Bull* 30 295–307. 10.1007/s12264-013-1406-z 24590577 PMC5562652

[B13] Khan M. T. (2009). Molecular interactions of cholinesterases inhibitors using in silico methods: current status and future prospects. *N. Biotechnol.* 25 331–346. 10.1016/j.nbt.2009.03.008 19491049

[B14] Kim J. Shin K. Cha Y. Ban Y. H. Park S. K. Jeong H. S. (2020). Neuroprotective effects of human neural stem cells over-expressing choline acetyltransferase in a middle cerebral artery occlusion model. *J. Chem. Neuroanat*. 103:101730. 10.1016/j.jchemneu.2019.101730 31837389

[B15] Kim Y. R. Kim H. N. Ahn S. M. Choi Y. H. Shin H. K. Choi B. T. (2014). Electroacupuncture promotes post-stroke functional recovery via enhancing endogenous neurogenesis in mouse focal cerebral ischemia. *PLoS One* 9:e90000. 10.1371/journal.pone.0090000 24587178 PMC3933702

[B16] Konar A. Gupta R. Shukla R. K. Maloney B. Khanna V. K. Wadhwa R. (2019). M1 muscarinic receptor is a key target of neuroprotection, neuroregeneration and memory recovery by i-Extract from Withania somnifera. *Sci. Rep.* 9:13990. 10.1038/s41598-019-48238-6 31570736 PMC6769020

[B17] Lebois E. P. Thorn C. Edgerton J. R. Popiolek M. Xi S. (2018). Muscarinic receptor subtype distribution in the central nervous system and relevance to aging and Alzheimer’s disease. *Neuropharmacology* 136 362–373. 10.1016/j.neuropharm.2017.11.018 29138080

[B18] Lee B. Sur B. Shim J. Hahm D. H. Lee H. (2014). Acupuncture stimulation improves scopolamine-induced cognitive impairment via activation of cholinergic system and regulation of BDNF and CREB expressions in rats. *BMC Compl. Altern Med.* 14:338. 10.1186/1472-6882-14-338 25231482 PMC4180318

[B19] Li L. Li L. Zhang J. Huang S. Liu W. Wang Z. (2020). Disease Stage-Associated Alterations in Learning and Memory through the Electroacupuncture Modulation of the Cortical Microglial M1/M2 Polarization in Mice with Alzheimer’s Disease. *Neural. Plast.* 2020:8836173. 10.1155/2020/8836173 32908486 PMC7474773

[B20] Li X. Guo F. Zhang Q. Huo T. Liu L. Wei H. (2014). Electroacupuncture decreases cognitive impairment and promotes neurogenesis in the APP/PS1 transgenic mice. *BMC Compl. Altern Med.* 14:37. 10.1186/1472-6882-14-37 24447795 PMC3907495

[B21] Lin R. Li L. Zhang Y. Huang S. Chen S. Shi J. (2018). Electroacupuncture ameliorate learning and memory by improving N-acetylaspartate and glutamate metabolism in APP/PS1 mice. *Biol. Res.* 51:21. 10.1186/s40659-018-0166-7 29980225 PMC6034239

[B22] Liu W. Zhuo P. Li L. Jin H. Lin B. Zhang Y. (2017). Activation of brain glucose metabolism ameliorating cognitive impairment in APP/PS1 transgenic mice by electroacupuncture. *Free Radic. Biol. Med.* 112 174–190. 10.1016/j.freeradbiomed.2017.07.024 28756309

[B23] Moon C. M. Kim B. C. Jeong G. W. (2016). Effects of donepezil on brain morphometric and metabolic changes in patients with Alzheimer’s disease: A DARTEL-based VBM and ^1^H-MRS. *Magn. Reson. Imag.* 34 1008–1016. 10.1016/j.mri.2016.04.025 27131829

[B24] Nelson A. Mooney R. (2016). The Basal Forebrain and Motor Cortex Provide Convergent yet Distinct Movement-Related Inputs to the Auditory Cortex. *Neuron* 90 635–648. 10.1016/j.neuron.2016.03.031 27112494 PMC4866808

[B25] Oomen C. A. Hvoslef-Eide M. Heath C. J. Mar A. C. Horner A. E. Bussey T. J. (2013). The touchscreen operant platform for testing working memory and pattern separation in rats and mice. *Nat. Protoc.* 8 2006–2021. 10.1038/nprot.2013.124 24051961 PMC3982138

[B26] Orta-Salazar E. Aguilar-Vázquez A. Martínez-Coria H. Luquín-De Anda S. Rivera-Cervantes M. Beas-Zarate C. (2014). REST/NRSF-induced changes of ChAT protein expression in the neocortex and hippocampus of the 3xTg-AD mouse model for Alzheimer’s disease. *Life* S*ci.* 116 83–89. 10.1016/j.lfs.2014.09.013 25261598

[B27] Parizkova M. Lerch O. Andel R. Kalinova J. Markova H. Vyhnalek M. (2020). Spatial Pattern Separation in Early Alzheimer’s Disease. *J. Alzheimers Dis.* 76 121–138. 10.3233/JAD-200093 32444544

[B28] Park S. Lee J. H. Yang E. J. (2017). Effects of Acupuncture on Alzheimer’s Disease in Animal-Based Research. *Evid Based Compl. Alternat. Med.* 2017:6512520. 10.1155/2017/6512520 29234418 PMC5635287

[B29] Phan A. Suschkov S. Molinaro L. Reynolds K. Lymer J. M. Bailey C. D. (2015). Rapid increases in immature synapses parallel estrogen-induced hippocampal learning enhancements. *Proc. Natl. Acad. Sci. U.S.A.* 112 16018–16023. 10.1073/pnas.1522150112 26655342 PMC4703013

[B30] Rekik K. Francés B. Valet P. Dray C. Florian C. (2017). Cognitive deficit in hippocampal-dependent tasks in Werner syndrome mouse model. *Behav. Brain Res.* 323 68–77. 10.1016/j.bbr.2017.01.034 28119126

[B31] Richetin K. Petsophonsakul P. Roybon L. Guiard B. P. Rampon C. (2017). Differential alteration of hippocampal function and plasticity in females and males of the APPxPS1 mouse model of Alzheimer’s disease. *Neurobiol. Aging* 57 220–231. 10.1016/j.neurobiolaging.2017.05.025 28666707

[B32] Sahay A. Scobie K. N. Hill A. S. Carroll C. M. Kheirbek M. A. (2011). Increasing adult hippocampal neurogenesis is sufficient to improve pattern separation. *Nature* 472 466–470. 10.1038/nature09817 21460835 PMC3084370

[B33] Saifullah M. Komine O. Dong Y. Fukumoto K. Sobue A. Endo F. (2020). Touchscreen-based location discrimination and paired associate learning tasks detect cognitive impairment at an early stage in an App knock-in mouse model of Alzheimer’s disease. *Mol. Brain* 13:147. 10.1186/s13041-020-00690-6 33183323 PMC7664057

[B34] Schliebs R. Arendt T. (2011). The cholinergic system in aging and neuronal degeneration. *Behav. Brain Res.* 221 555–563. 10.1016/j.bbr.2010.11.058 21145918

[B35] Sharma K. (2019). Cholinesterase inhibitors as Alzheimer’s therapeutics (Review). *Mol. Med. Rep*. 20, 1479–1487. 10.3892/mmr.2019.1037 31257471 PMC6625431

[B36] Stevenson R. F. Reagh Z. M. Chun A. P. Murray E. A. Yassa M. A. (2020). Pattern Separation and Source Memory Engage Distinct Hippocampal and Neocortical Regions during Retrieval. *J. Neurosci.* 40 843–851. 10.1523/JNEUROSCI.0564-19.2019 31748377 PMC6975302

[B37] Sultzer D. L. (2018). Cognitive ageing and Alzheimer’s disease: the cholinergic system redux. *Brain* 141 626–628. 10.1093/brain/awy040 30753416

[B38] Wang W. W. Xie C. L. Lu L. Zheng G. Q. (2014). A systematic review and meta-analysis of Baihui (GV20)-based scalp acupuncture in experimental ischemic stroke. *Sci. Rep.* 4:3981. 10.1038/srep03981 24496233 PMC5379241

[B39] Wang Y. Y. Yu S. F. Xue H. Y. Li Y. Zhao C. Jin Y. H. (2020). Effectiveness and Safety of Acupuncture for the Treatment of Alzheimer’s Disease: A Systematic Review and Meta-Analysis. *Front. Aging Neurosci.* 12:98. 10.3389/fnagi.2020.00098 32435187 PMC7218057

[B40] Xiao Y. S. (2017). Effects of Huangjing Dilong extracts on M-AchR expression in senile dementia rat’s brain. *Pharmacol. Clin. Chin. Mat. Med.* 33 110–114.

[B41] Yan H. Pang P. Chen W. Zhu H. Henok K. A. Li H. (2018). The Lesion Analysis of Cholinergic Neurons in 5×FAD Mouse Model in the Three-Dimensional Level of Whole Brain. *Mol. Neurobiol.* 55 4115–4125. 10.1007/s12035-017-0621-4 28597200

[B42] Yu C. C. Du Y. J. Wang S. Q. Liu L. B. Shen F. Wang L. (2020). Experimental Evidence of the Benefits of Acupuncture for Alzheimer’s Disease: An Updated Review. *Front. Neurosci.* 14:549772. 10.3389/fnins.2020.549772 33408601 PMC7779610

[B43] Zaletel I. Schwirtlich M. Peroviæ M. Jovanoviæ M. Stevanoviæ M. Kanazir S. (2018). Early Impairments of Hippocampal Neurogenesis in 5xFAD Mouse Model of Alzheimer’s Disease Are Associated with Altered Expression of SOXB Transcription Factors. *J. Alzheimers Dis.* 65 963–976. 10.3233/JAD-180277 30103323

[B44] Zeng Q. Zheng M. Zhang T. He G. (2016). Hippocampal neurogenesis in the APP/PS1/nestin-GFP triple transgenic mouse model of Alzheimer’s disease. *Neuroscience* 314 64–74. 10.1016/j.neuroscience.2015.11.054 26639620

[B45] Zhan J. Komal R. Keenan W. T. Hattar S. Fernandez D. C. (2019). Non-invasive Strategies for Chronic Manipulation of DREADD-controlled Neuronal Activity. *J. Vis. Exp.* 150:59439. 10.3791/59439 31498301 PMC6818240

[B46] Zhang H. Y. Watson M. L. Gallagher M. Nicolle M. M. (2007). Muscarinic receptor-mediated GTP-Eu binding in the hippocampus and prefrontal cortex is correlated with spatial memory impairment in aged rats. *Neurobiol. Aging* 28 619–626. 10.1016/j.neurobiolaging.2006.02.016 16600436

[B47] Zhang Q. Y. Wang Z. J. Miao L. Wang Y. Chang L. L. Guo W. (2019). Neuroprotective Effect of SCM-198 through Stabilizing Endothelial Cell Function. *Oxid. Med. Cell Longev.* 2019:7850154. 10.1155/2019/7850154 31827699 PMC6885260

[B48] Zhao L. X. Chen M. W. Qian Y. Yang Q. H. Ge Y. H. Chen H. Z. (2019). M1 Muscarinic Receptor Activation Rescues β-Amyloid-Induced Cognitive Impairment through AMPA Receptor GluA1 Subunit. *Neuroscience* 408 239–247. 10.1016/j.neuroscience.2019.04.007 30981860

[B49] Zhu H. Yan H. Tang N. Li X. Pang P. Li H. (2017). Impairments of spatial memory in an Alzheimer’s disease model via degeneration of hippocampal cholinergic synapses. *Nat. Commun.* 8:1676. 10.1038/s41467-017-01943-0 29162816 PMC5698429

